# MaxEnt brings comparable results when the input data are being completed; Model parameterization of four species distribution models

**DOI:** 10.1002/ece3.9827

**Published:** 2023-02-17

**Authors:** Mohsen Ahmadi, Mahmoud‐Reza Hemami, Mohammad Kaboli, Farzin Shabani

**Affiliations:** ^1^ Department of Natural Resources Isfahan University of Technology Isfahan Iran; ^2^ Department of Environmental Sciences, Faculty of Natural Resources University of Tehran Karaj Iran; ^3^ Department of Biological and Environmental Sciences College of Arts and Sciences, Qatar University Doha Qatar

**Keywords:** habitat suitability, MaxEnt, model tuning, spatially imbalanced‐biased

## Abstract

Species distribution models (SDMs) are practical tools to assess the habitat suitability of species with numerous applications in environmental management and conservation planning. The manipulation of the input data to deal with their spatial bias is one of the advantageous methods to enhance the performance of SDMs. However, the development of a model parameterization approach covering different SDMs to achieve well‐performing models has rarely been implemented. We integrated input data manipulation and model tuning for four commonly‐used SDMs: generalized linear model (GLM), gradient boosted model (GBM), random forest (RF), and maximum entropy (MaxEnt), and compared their predictive performance to model geographically imbalanced‐biased data of a rare species complex of mountain vipers. Models were tuned up based on a range of model‐specific parameters considering two background selection methods: random and background weighting schemes. The performance of the fine‐tuned models was assessed based on recently identified localities of the species. The results indicated that although the fine‐tuned version of all models shows great performance in predicting training data (AUC > 0.9 and TSS > 0.5), they produce different results in classifying out‐of‐bag data. The GBM and RF with higher sensitivity of training data showed more different performances. The GLM, despite having high predictive performance for test data, showed lower specificity. It was only the MaxEnt model that showed high predictive performance and comparable results for identifying test data in both random and background weighting procedures. Our results highlight that while GBM and RF are prone to overfitting training data and GLM over‐predict nonsampled areas MaxEnt is capable of producing results that are both predictable (extrapolative) and complex (interpolative). We discuss the assumptions of each model and conclude that MaxEnt could be considered as a practical method to cope with imbalanced‐biased data in species distribution modeling approaches.

## Graphical abstract

The input data for species distribution modeling (SDM) are always completing, and data of the unknown and range‐restricted species are mostly spatially imbalanced‐biased. Model parameterization is necessary to improve the predictive performance of the species distribution models. The MaxEnt model provides reproducible results when the input data are being developed.

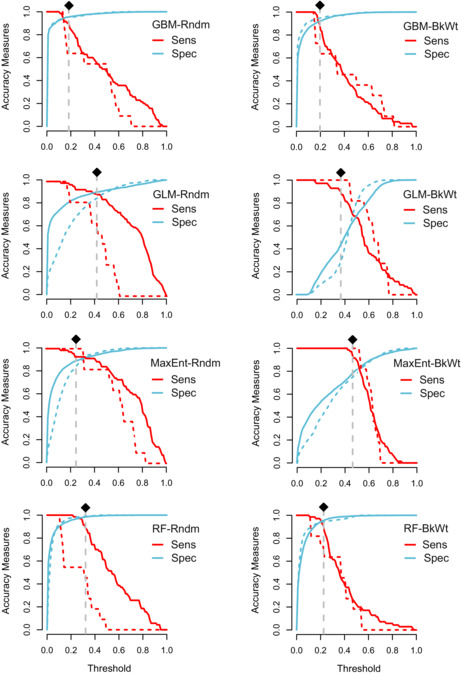

## 1 INTRODUCTION

Species distribution models (SDMs) by combining data of species occurrence and environmental variables are operative tools to understand the dynamics of biodiversity distribution in space and time. A wealth of literature exists on the utility of SDMs all aiming at explaining, predicting, and projecting species distribution (Araújo et al., 2019). In particular identifying geographic distribution and most effective variables in different geographic scales (Brito et al., 2009; Hemami et al., 2018; Salinas‐Ramos et al., 2021), assessing conservation coverage and efficiency of protected areas (Farhadinia et al., 2015; Lentini & Wintle, 2015; Zupan et al., 2014), predicting the biological invasion of alien species (Bosso et al., 2022; Thuiller et al., 2005; Tingley et al., 2014), climate change‐induced range shifts (Lorestani et al., 2022; Thuiller et al., 2011; Yousefi et al., 2015), and combining its results with phylogenetic analyses to explore species evolutionary history (Ahmadi et al., 2018; Ahmadzadeh et al., 2016; Boucher et al., 2015; Saladin et al., 2019) are among the most widely‐used aspects of SDMs.

In general, a variety of SDMs with different algorithms has been developed, which may lead to different results for a target species (Elith & Graham, 2009; Merow et al., 2014). Consequently, model manipulation and comparing their results have been the subject of a significant amount of debate and research (Elith et al., 2006; Shabani et al., 2016; Valavi et al., 2022; Wisz et al., 2008). According to Araújo et al. (2019), four aspects of SDMs determine the quality of the resulting model, including response variable (i.e., occurrence records of the species), predictor variables, model building, and model evaluation. An SDM is a process of modeling and prediction, thus, contains levels of uncertainty that rises from each of the above‐mentioned aspects.

At each level, solutions have been proposed to increase the quality of the data and reduce the negative effects of uncertainty in the output models. For example, in the first step, improving the sampling design can reduce bias and inaccuracy in the geographical distribution of the collected data (Araújo & Guisan, 2006; Chauvier et al., 2021). At this level, ensuring that the collected data correctly represent the actual distribution of the species (Guillera‐Arroita et al., 2015; Tessarolo et al., 2014) and that the scale of modeling and independent variables are consistent with sampling precision (Chauvier et al., 2022; Guisan et al., 2007; Wiens et al., 2009), and reducing unbiased recognition of the taxonomy of the species (Lorestani et al., 2022; Rocchini et al., 2011) improve results of an SDM analysis.

An essential hypothesis of statistical methods is that recorded data are independent (i.e., randomly allocated samples with independent distribution), requiring that the entire area of interest is randomly or systematically sampled. In practice, available data on the species distribution is spatially biased toward areas easily assessed and/or better surveyed (Araújo & Guisan, 2006; Boria et al., 2014). A different strategy and intensity of sampling cause uneven distribution of recorded data, inconsistent with the real spatial ecology of the target species. This spatial bias may result in spatial clumping, which in turn, leads to the over‐representation of areas with a higher density of input data in the model. This can lead to spatial autocorrelation (SAC) of occurrence points (Dormann et al., 2007) that inflates model accuracy (Veloz, 2009), and misleads parameter estimates (Kramer‐Schadt et al., 2013).

In general, manipulating the input data (Chauvier et al., 2021; Elith et al., 2010), and parameterizing the modeling method (Fithian et al., 2015; Muscarella et al., 2014) are two strategies that have been used to take into account the bias in SDM efforts. In particular, the bias caused by spatial autocorrelation could be reduced by spatial filtering (Boria et al., 2014; Kramer‐Schadt et al., 2013) and background weighting schemes, the latter is also called “target‐group background” (Elith et al., 2010; Phillips et al., 2009). This method reduces the bias in a way that favors areas densely sampled over sparsely sampled areas (Phillips et al., 2009; Shabani et al., 2016).

On the contrary, parameterizing SDMs to obtain a fine‐tuned model is an aspect that has been poorly considered. In almost all cases, the default settings are being used to perform SDMs, especially for complex machine learning ones (Kass et al., 2021). In addition to increase the possibility of overfitting caused by noisy data (Merow et al., 2014), the default setting decreases model transferability during the projection to a novel environment (Guevara et al., 2018). Applying different levels of complexity and evaluating the balance between the bias and variance of models allow to find the optimal model with a justified level of complexity (Araújo et al., 2019; Radosavljevic & Anderson, 2014). However, among the few attempts to parameterize SDMs are tuning the best combination of the primary models to a final ensemble model (Kindt, 2018; Thuiller et al., 2009) or applying a set of input parameters to fine‐tune the MaxEnt model, e.g., the package ENMeval (Muscarella et al., 2014). The development of new tools, for example, h2o platform (Candel et al., 2016) or caret package (Kuhn, 2021) can bring SDM parameterization into a new focus. However, a holistic effort in which a wider range of species distribution models are fine‐tuned has so far, to our knowledge, not been implemented in this arena of research.

Using SDMs is particularly pragmatic for scarce species as the results of these methods provide valuable information for their conservation implementations (Farhadinia et al., 2015; Franklin, 2010) and for identifying target areas for future sampling (Galante et al., 2018). Notwithstanding, data on scarce species mostly suffer spatial bias due to imbalanced sampling surveys (El‐Gabbas & Dormann, 2018a; Rebelo & Jones, 2010). In this research, we evaluated the performance of different SDMs to identify new populations of the rare species of the genus *Montivipera* in the mountains of Iran, Turkey, and Armenia. From a phylogeographic point of view, the species of this genus due to their rapid ratio of speciation in the recent evolutionary scales show interesting forms of neo‐endemism in the Near and Middle East (Behrooz et al., 2018; Stümpel et al., 2016). This genus consists of two complex groups of species, *M. xanthina* complex and *M. raddei* complex. In this research, we focused on *M. raddei* complex (MRC) distributed across mountainous landscapes of northeastern Turkey, Armenia, and Iran. The northern populations of the MRC are well‐described and all their potential habitats are geographically well‐sampled. On the contrary, the southern ranges in Iran across the Zagros Mountains have not proportionally been sampled and some new populations of these species plus a newly defined species have just recently been identified (Behrooz et al., 2018). Accordingly, the data of the species distribution due to the different intensity and quality of sampling is geographically imbalanced‐biased. Here we integrated model parameterization and data manipulation to evaluate the proficiency of four correlative SDMs including generalized linear models (GLM), gradient boosting model (GBM), random forest (RF), and maximum entropy (MaxEnt) for locating recently discovered *Montivipera* populations. Models were fine‐tuned based on their intrinsic parameters, and the input data were bias‐corrected by implementing a background weighting procedure. We then compared the results with models of random background procedure given the new populations as out‐of‐bag data to test the models. In addition to AUC and TSS, as two commonly‐used measures of model accuracy, we also depicted the accuracy of the models across the entire gradient of suitability thresholds to provide a better understanding of the model's demeanor in classifying spatially imbalanced‐biased data of the species.

## 2 MATERIALS AND METHODS

### 2.1 Montivipera occurrence points

**FIGURE 1:**
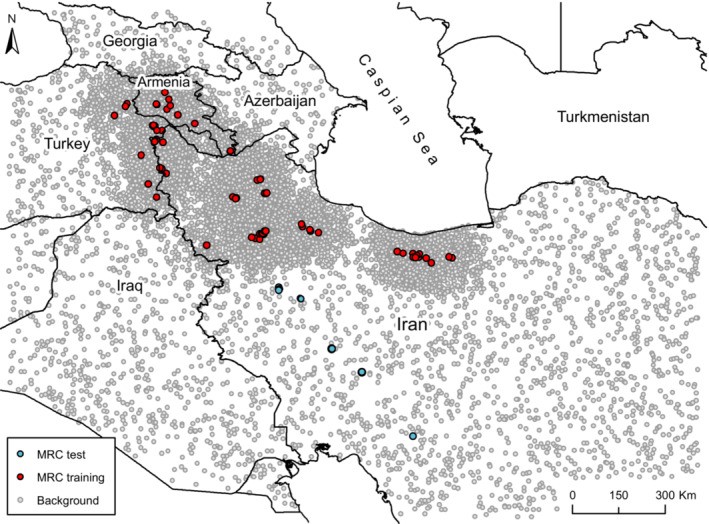
Geographic distribution of MRC occurrence and background‐weighted points. Background‐weighted points were allocated based on a probability distribution of a density raster of training occurrence points.

In the current research, we used the most complete dataset of MRC distribution across mountainous landscapes of Iran, eastern Turkey, and southern Armenia (Figure 1). We collected the presence points (*n* = 91) throughout the entire range of the species based on direct field research, other herpetologists' field studies, and literature review. All the presence points were assessed based on the IUCN range map and spatial outliers (especially those from the literature) were excluded. Moreover, to cope with the probable spatial autocorrelation of the occurrence points we removed repeated points within a buffer of a 5‐km radius, which remained us 82 occurrence points. The coverage of all collected records indicates apparent signs of spatial bias toward north and northwestern Iran, eastern Turkey, and Armenia, and sparse sampling across the Zagros Mountains in western Iran, where new localities were just recently recorded. Therefore, the occurrence points were split into training and testing data, using records from northern parts for training and keeping the newly identified presence data from southern parts as independent evaluation data.

### 2.2 SDM development

Models were performed using data of species presence points and explanatory variables. Two sets of variables were considered: climate and vegetation. For climatic variables, we focused on four primary variables describing the annual mean and variability of climatic conditions including mean annual temperature (meananultmp), annual precipitation (anulprc), temperature seasonality (tmpseas), and precipitation seasonality (precseas) all downloaded from WorldClim dataset (Hijmans et al., 2005). By reflecting adaptation to climatic variability, these variables represent important environmental constraints on *Montivipera*'s distribution, niche evolution, and adaptability (Ahmadi et al., 2021). In addition to climatic variables, the habitat suitability of mountain vipers is also influenced by vegetation (Yousefi et al., 2015). For the vegetation variable, we used the enhanced vegetation index (EVI) of the MODIS products (MOD13A3) and adopted the same variability in climatic variables for vegetation, i.e., mean annual EVI and EVI seasonality. To do so, we downloaded monthly‐provided 1‐kilometer‐resolution MOD13A3 for 2015 from EarthExplorer dataset (https://earthexplorer.usgs.gov), extracted EVI bands in ENVI version 5.1, mosaicked them to cover the entire study area in one scene, and calculated the annual mean and standard deviation of the 12 monthly‐EVI rasters. We used EVI instead of commonly‐used NDVI, because of its potential to minimize canopy background variations and maintain sensitivity over dense vegetation conditions (Jiang et al., 2008). The EVI also copes better with residual atmosphere contamination caused by smoke and sub‐pixel thin clouds. Before SDM analysis, using the variance inflation factor (VIF) in “usdm” package (Naimi, 2015), we checked the multicollinearity of variables.

We focused on four SDM methods, GLM, GBM, RF, and MaxEnt, because of their prevalence, well‐performance, and approval over other methods (Elith & Graham, 2009; Phillips et al., 2006; Shabani et al., 2016), all implemented in R environment v 3.5.2. We first split MRC occurrence points to training and test data and followed a cross‐validation scheme on the training dataset to fit the models. This cross‐validated scheme of training data was kept constant for tuning the preliminary models and running the final version of the models. GLM, GBM, and RF were tuned up using the “caret” package (Kuhn, 2021) with considering different model‐specific parameters, and the best‐fitted model across the then folds was identified according to their ROC scores. The fine‐tuned model with the highest accuracy was then used to generate the habitat suitability maps and to predict the test dataset. The GLM was performed using simple and quadratic terms of explanatory variables and the model selection was based on a stepwise AIC selection procedure. GBM was fitted with allowing the maximum number of trees up to 2000, with three learning rates (i.e., shrinkage; 0.001, 0.01, 0.1), three interaction depths (i.e., complexity of the tree, maximum nodes per tree; 1, 3, and 5), and three values for subsampling fraction (i.e., bag fraction; 0.5, 0.65, and 0.8). The RF model was fitted with number of trees (ntrees) 500 and 1000, number of variables randomly selected at each split (i.e., mtry) 1 to 5, and node size 1 and 5. The MaxEnt model was tuned up using the package ENMeval (Muscarella et al., 2014) allowing five combinations of feature types (fc = L, LQ, LQH, LQHP, and LQHPT) and regularization multiplier (rm) of 0.5, 1, 1.5, and 2. The best‐fitted parameters for each model were then used to predict the environmental layers and to generate the corresponding habitat suitability maps. Again, the generation of habitat suitability maps was carried on given the constant 10‐fold cross‐validation of the occurrence points, meaning that 10 habitat suitability map was predicted for each model. The final ensemble habitat suitability map of the four SDM algorithms was generated based on a proportionally weighted average of the obtained AUC score of the 10 repetitions.

To address the purpose of our study, background data were selected in two different ways including random and background weighting. For random we selected 10,000 background points spatially at random leaving cells with MRC occurrence points. To create background weighting data by generating a weighting surface we gave prominence to those areas having less geographical proximity to others. Following Elith et al. (2010) we first generated a density raster map from the occurrence points and then allocated 10,000 background points regarding its probability distribution (Figure 1). This method copes with the bias caused by the spatially imbalanced‐biased data in a way that favors occurrence points of severely sampled areas over those of sparsely sampled areas (Shabani et al., 2019). Of the 82 occurrence points of MRC, we used 12 newly sampled records as out‐of‐bag data to test models' performance. We used the area under the curve (AUC) of the receiver‐operating characteristic (ROC) plot to assess the discrimination capacity of models. AUC combines specificity and sensitivity (Fielding & Bell, 1997) and, thus, neglects the relative costs of errors of omission and commission (Jiménez‐Valverde, 2012). Therefore, we also computed true statistic skill (TSS) as a threshold‐dependent measure of classification accuracy calculated as sensitivity + specificity – 1. We used the package “PresenceAbsence” to evaluate the performance of the models and the threshold “10 percentile of training suitability” (Ahmadi et al., 2020; Rezaei et al., 2022) was set to calculate the threshold‐dependent measures. In Addition to these traditionally‐used metrics, which give an absolute measure of the model performance, we plotted the sensitivity and specificity of the models against an ascending gradient of 100 thresholds to obtain more informative inferences on the models' predictive performance.

## 3 RESULTS

Checking variables' multicollinearity indicated no VIF > 6 (Table 1), hence, we used all six explanatory variables in the SDM analysis. In total, for each of the random and background weighting schemes we fitted 128 preliminary models based on the 10 cross‐validated folds of the training occurrence points. Since the GLM is inherently a simple algorithm, for this method only one set of parameters was trained. Although for both the training and test datasets, the AUC and TSS of this model in the background weighting scheme decreased in comparison with the random scheme (Tables 2 and 3), it successfully classified all the test data, i.e., sensitivity = 1. Totally, GLM obtained AUC of 0.92 and 0.89 and TSS of 0.66 and 0.65 for random and background weighting schemes, respectively (Table 2). From the multiple combination of the GBM parameters, for the random background selection, a model characterized by shrinkage = 0.01, interaction depth = 5, and ntrees = 1800 showed the highest ROC value (ROC = 1, sensitivity = 1, and specificity = 0.85). For background weighting, the fine‐tuned GBM model (ROC = 1, sensitivity = 1, and specificity = 0.64) had shrinkage = 0.01, interaction depth = 5, and ntrees = 2000. Although for both training and test datasets the AUC and TSS of this model were almost equal (AUC = 0.976 and 0.971, and TSS = 0.59 and 0.58 for random and background weighting schemes, respectively), it lost the ability to truly predict presence points, i.e., sensitivity, compared with other models (Table 2). For the RF model, the fine‐tuned model of both random and background weighting schemes was characterized by ntrees = 1000, and nodesize = 1. However, the mtry was 2 and 3 for random and background weighting schemes, respectively. Similar to the GBM, RF obtained almost equal AUC and TSS scores for both training and test datasets (AUC = 0.97 and 0.96 and TSS = 0.45 and 0.58 for random and background weighting schemes, respectively), but the sensitivity of this model was low (Table 2). For the MaxEnt model, the best‐fitted model with the highest AIC_w_ of the ENMeval analysis obtained rm 0.5 and 1.5, and fc LQ and LQHP for random and background weighting schemes, respectively. In the final habitat suitability maps of the MaxEnt model, the test data had AUC 0.93 and 0.95 and TSS 0.66 and 0.80 for random and background weighting schemes, respectively (Table 2). Overall, the highest TSS score of the test data was obtained in the MaxEnt model that was fitted based on the background weighting scheme (Table 2).

**TABLE 1:** The results of variables' variance inflation factor (VIF) calculated to assess multicollinearity between them.

Variable	VIF
Annual mean temperature	5.7
Temperature seasonality	3.1
Annual precipitation	5.2
Precipitation seasonality	3.9
Annual mean EVI	5.4
EVI seasonality	5.8

Abbreviations: EVI, enhanced vegetation index.

**TABLE 2:** The average performance of the models across 10 cross‐validated folds of the training dataset calculated for random and background weighting (BkWt) schemes. The threshold‐dependent measures were calculated given a 10 percentile of suitability score at the training occurrence points.

	Sensitivity		Specificity		AUC		TSS	
	Random	BkWt	Random	BkWt	Random	BkWt	Random	BkWt
GBM	0.9	0.9	0.955	0.921	0.98	0.964	0.855	0.821
GLM	0.9	0.9	0.894	0.681	0.947	0.838	0.794	0.581
MaxEnt	0.9	0.9	0.887	0.683	0.947	0.828	0.787	0.583
RF	0.9	0.9	0.984	0.956	0.994	0.979	0.884	0.856

**TABLE 3:** Results of the model evaluation for random and background weighting (BkWt) schemes. Models were evaluated based on the 12 newly sampled occurrence points of the MRC. The threshold‐dependent measures were calculated given a 10 percentile of suitability score at the training occurrence points.

	Sensitivity		Specificity		AUC		TSS	
	Random	BkWt	Random	BkWt	Random	BkWt	Random	BkWt
GBM	0.636	0.636	0.953	0.947	0.976	0.971	0.589	0.583
GLM	0.818	1	0.84	0.657	0.917	0.893	0.658	0.647
MaxEnt	0.818	1	0.843	0.804	0.927	0.949	0.661	0.804
RF	0.455	0.636	0.991	0.945	0.97	0.957	0.446	0.581

**FIGURE 2:**
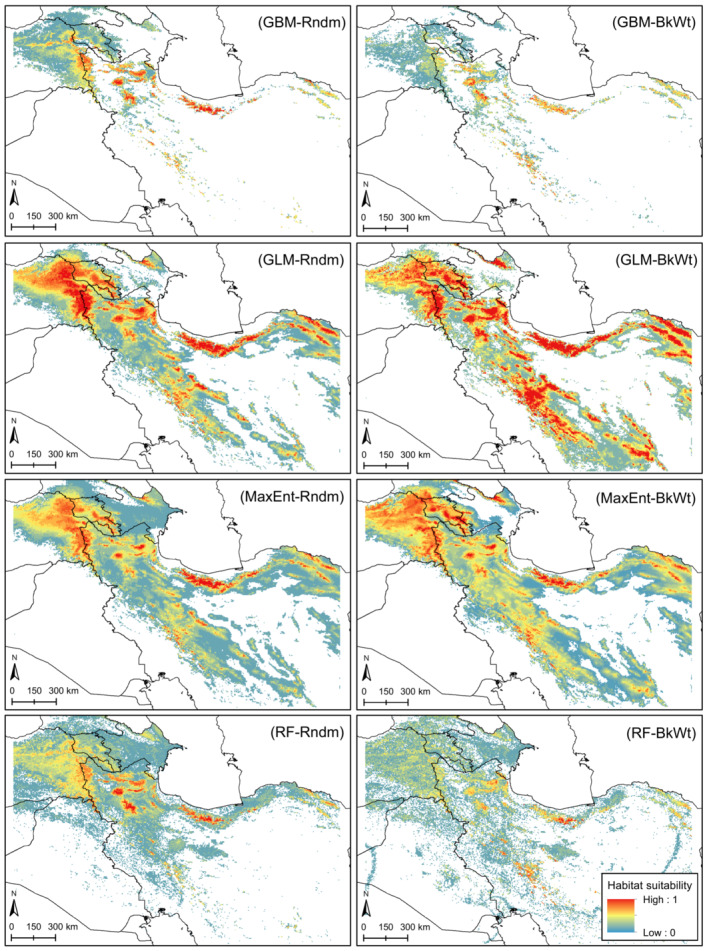
Habitat suitability map of the tuned‐up models derived from random (Rndm) and background weighting (BkWt) schemes.

**FIGURE 3:**
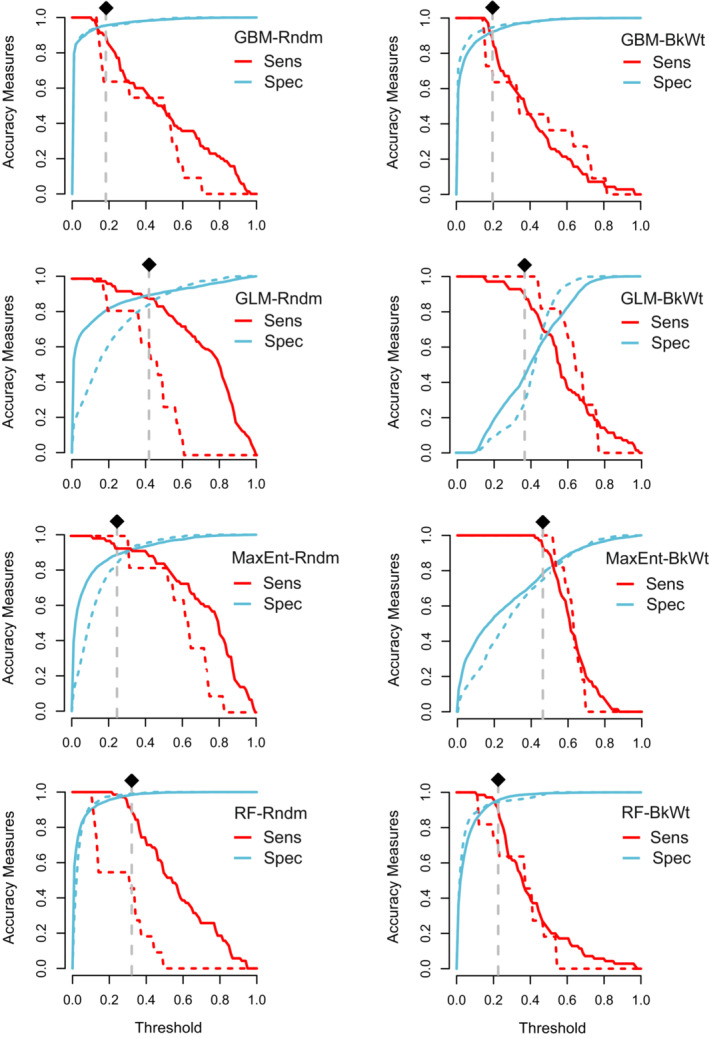
Variation of sensitivity (Sens); the proportion of correctly predicted presence data, and specificity (Spec); the proportion of correctly predicted pseudoabsence data, across a gradient of suitability thresholds. Dashed lines indicate test data. Rndm, random and BkWt, background weighting schemes. The black diamonds indicate the corresponding threshold of the 10‐percentile suitability of the training dataset.

**FIGURE 4:**
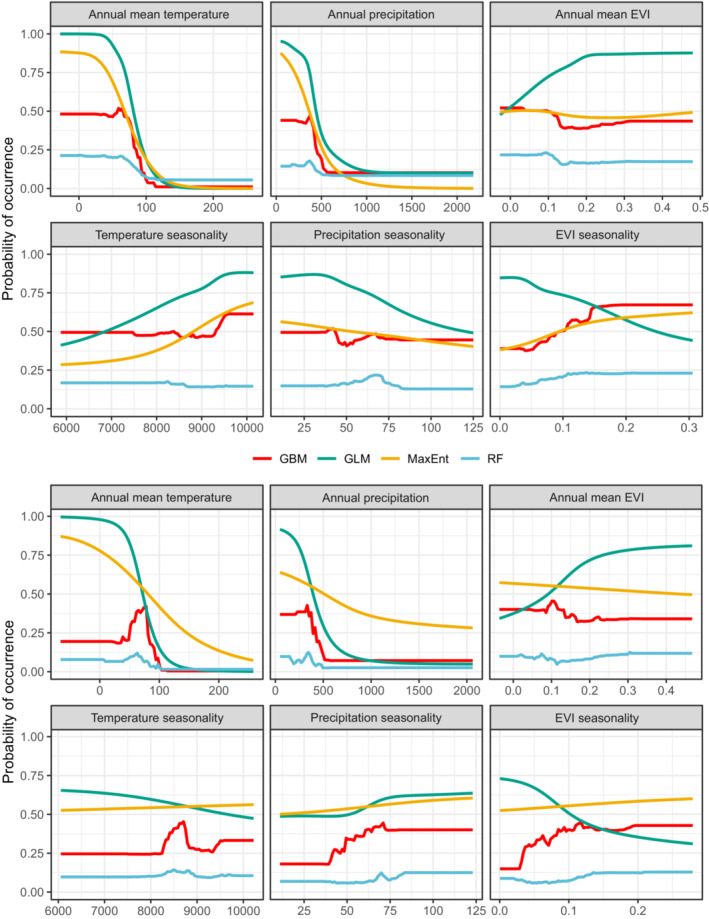
Response curve of the explanatory variables derived from models obtained based on the random (top) and background weighting (bottom) schemes. Models were fitted in biomod2 package.

The predicted suitability maps are shown in Figure 2. We found a good consistency between the patterns of occurrence points and suitable habitats. Comparing the spatial pattern of suitable habitats in random and background weighting methods showed that all models represented different results except for MaxEnt model in which comparable results were obtained (Figure 2). Accordingly, we calculated the correlation coefficient between the two background selection schemes of the four SDM methods revealing that the highest correlation was obtained for MaxEnt model (*r* = 0.85), followed by GLM (*r* = 0.61), GBM (*r* = 0.45), and RF (*r* = 0.42). These findings were also confirmed by sensitivity and specificity graphs (Figure 3). We found that while the capacity of GBM and RF to predict the training and test background points (i.e., models' specificity) was maintained excellent even at higher thresholds, their capability to predict presence data (i.e., models' sensitivity) was reduced at lower thresholds. On the contrary, GLM and MaxEnt models showed good performances to predict presence data but lost their capacity to classify background data at lower thresholds especially in the background weighting scheme (Figure 3). The comparison of the response curves of the variables between the two background selection schemes indicated an identical pattern; however, for GBM and RF, the response curves of the background weighting scheme were more rugged compared with the smoother variation in the random background selection scheme (Figure 4).

## 4 DISCUSSION

In the present study, the performance of different models in predicting spatially‐biased occurrence data was evaluated. For this purpose, modeling was performed based on four SDMs. A variety of correlative distribution models have been emerged in recent decades and their performance has been compared in numerous studies (Elith & Graham, 2009; Merow et al., 2014; Valavi et al., 2022). These methods, however, are challenged by a number of methodological problems that make their comparison controversial. Among these issues are providing a balance between goodness‐of‐fit and model complexity (Araújo et al., 2019; Warren & Seifert, 2011), the spatial bias of the input data, and manipulating them for evaluating model performance (Chauvier et al., 2022; Hijmans, 2012; Phillips et al., 2009). Generally, for most SDMs, particularly for complex machine learning ones, using a set of default parameters has been recommended based on a comprehensive model tuning [for example see Phillips & Dudík, 2008 for the MaxEnt and Elith et al., 2008 for boosted regression trees]. However, a blindfold utilization of them may come up with a poorly performing model (Muscarella et al., 2014). Equally important, is the probable SAC in the input occurrence points, which may result in an inflated score of the metrics used for model valuation (Dormann et al., 2007; Radosavljevic & Anderson, 2014). Another problem is the paucity of operative procedures for tuning SDMs that are efficiently effortless and time‐saving (Valavi et al., 2022). Consequently, in most SDM efforts, while the species, scale, and area of interest are different, the modeling relies on the default setting of the related model. In the current research, we employed a novel approach that on one hand applied model‐specific parameterization, and on the other hand, manipulated a spatially imbalanced‐biased input data to cope with the above‐mentioned issues.

### 4.1 Models comparison: Simple regression vs. complex machine learning

In general, the results showed that the models have high predictive performance based on AUC and TSS values. However, the geographical pattern of the predicted suitable habitats among models was different. For example, the area of suitable habitats was greater in GLM in comparison with other SDMs. Accordingly, for this model lower values of specificity were observed across the different gradients of suitability thresholds (red curves in Figure 3). This is intrinsically due to the simple regression‐based nature of this model in which a basic assumption is the normality of the error distribution and its constant variance (Osborne & Waters, 2002). Thus, those data that are not used as training are well‐fitted in the predicted model. On the other hand, this makes GLM prone to over‐prediction across nonsampled areas leading to lower specificity, i.e., true negative rate, of this model.

On the contrary, we found the best training sensitivity and specificity for decision‐tree‐based methods, i.e., GBM and RF. These models, due to the automatic promotion caused by the model learner, attempt to improve task classification, as much as possible, leading to the highest discrimination capacity of training presence/absence dataset (De'ath & Fabricius, 2000; Hastie et al., 2009). In our case, the results indicated that the RF models for both random and background weighting procedures, while excellently classified the training dataset, failed to predict out‐of‐bag ones. Fundamentally, RF and GBM depend on bagging and boosting algorithms of the tree learner, respectively (Elith et al., 2008; Hastie et al., 2009). In RF the bagging algorithm, also called bootstrap aggregation, allows the tree learner repeatedly select a random sample with replacement of the training set and fits trees to these samples (Breiman, 2001). The trees in RF are run in parallel and there is no interaction between them while the trees are built. Once all the trees are built, then an average is taken across all the trees' predictions (Cutler et al., 2012). Conversely, the trees in boosting algorithms, e.g., GBM, are trained sequentially, and accordingly, weaker results are boosted or reweighted over many iterations to have the learner focus more on areas it got wrong, and less on those observations that were correct (Hastie et al., 2009). The risk of deeply‐grown trees in random forests comes at the expense of overfitting the training dataset in which predictions have low bias but high variances. In our modeling approach, we grew the RF model to 1000 trees, which is reasonable and comparable with similar SDM efforts (Breiman, 2001; Shabani et al., 2016). Although based on a cross‐validated splitting of the training data we fine‐tuned the RF model, it appears that this model still suffers from high variance. In fact, the strict classification algorithm of the RF model is prone to return low capabilities in predicting out‐of‐bag datasets (Elith et al., 2008; Merow et al., 2014). Accordingly, the use of this method in extrapolating predictions should be taken into account prudently.

Given the random background approach, our results highlight the excellent predictive performance of the GBM model in which true positive rate, i.e., sensitivity, and true negative rate, i.e., specificity, remained well‐justified across accumulative thresholds. In contrast to the paralleled bagging method of the RF, it is believed that the boosting manner of the tree learner in GBM reduces the probability of overfitting and allows well‐classifying out‐of‐bag samples (Elith et al., 2008; Shabani et al., 2016). However, our study revealed that fitting a GBM model based on a background weighting approach significantly reduces the predictive performance of this model for identifying out‐of‐bag data. This highlights the inefficiency of this method for being used in SDM efforts with imbalanced‐biased data where the primary goal is finding probability distribution over areas that are not comparably sampled.

Unlike the decision‐tree‐based methods, MaxEnt resulted in a comparable prediction based on both random and background weighting approaches. This was similar to the results of the GLM model except that for the latter true negative rates were misplaced at lower thresholds. As mentioned before, the normally‐distributed errors and no trends in residuals relative to the fitted values (Hardin & Hardin, 2007) allow the GLM to be interpretably efficient for predicting out‐of‐bag data. In MaxEnt, as a density estimator algorithm, the species distribution is represented by a probability distribution that is closest to uniform (Pathak et al., 2022; Phillips et al., 2006). This probability distribution is bounded by a set of constraints that are simple functions of the explanatory variables, called “features,” and derived from the species occurrence locations. The primary assumption of the MaxEnt model is that the mean of each feature is required to be close (within same error bounds) to the empirical average over the presence sites (Phillips & Dudík, 2008). From a general point of view, this constraint in MaxEnt could be assumed equivalent to the consistency of error variance in the GLM method, and as a consequence, bringing consistent results for out‐of‐bag data is also expectable in the MaxEnt model. In both GLM and MaxEnt models, a maximum likelihood is used to estimate a parametric exponential distribution of a linear combination of features (Phillips et al., 2004). Although GLM could be fitted by considering quadratic and interactive terms of the explanatory variables, more variation of feature types in MaxEnt allows for fitting more complex models (Phillips et al., 2006). More importantly, while GBM and RF as complex machine learning methods are more prone to overfitting (Valavi et al., 2022), the regularization multiplier in MaxEnt prevents the model to match the input data too closely. Altogether, our results highlight the efficiency of the MaxEnt model in using the benefits of different modeling methods to produce results that are both predictable (extrapolative) and complex (interpolative).

### 4.2 Approaches to evaluate model performance

Asymmetry in spatially‐biased model predictions also highlights the need to evaluate model performance using threshold‐dependent sensitivity (true positive rate) and specificity (true negative rate) in addition to threshold‐independent AUC. As criticized by Lobo et al. (2008) and Jiménez‐Valverde (2012), modeling goals and setting highly influence the appropriateness of the AUC for measuring the performance of a model. AUC inflates the number of false absence data (Lobo et al., 2008), and accordingly, over‐represents predictive performance for rare species (Phillips et al., 2009; Stolar & Nielsen, 2015), as in the case of *Montivipera* species. Moreover, being only a discrimination measure, AUC does not show goodness‐of‐fit, i.e., classification accuracy of the model, and consequently, a model with a high AUC value is not necessarily a well‐fitted one (Jiménez‐Valverde, 2012). For threshold‐dependent measures a critical trick is selecting the best suitability threshold at which the sensitivity and specificity of the resulted model are well‐balanced. Although several thresholds have been suggested to do this, for example, see Liu et al. (2005) as a review, a single suitability threshold provides only a cross‐section of the model performance and does not provide a comprehensive perception of the classification accuracy of the model across a gradient of suitability threshold. For example, the threshold at which the sensitivity is equal to the specificity, i.e., where their curves cross in Figure 3, is among the widely‐used suitability thresholds, nevertheless, the corresponding accuracy measure does not inclusively specify the performance of the model to classify presence and background data.

### 4.3 Limitations of the study

There are several ways to correct sampling bias, some of which cannot be used in cases where data is scarce. Spatial filtering may not be helpful when there are only a few presence points (Phillips et al., 2009). Decreasing presence points clumping reduces training sample size and, depending on the heterogeneity of the surrounding environment and the selected spatial resolution, it may drop some of the information on the species occupation sites. In addition to spatial filtering and background weighting, a third method called model‐based bias correction has been used (El‐Gabbas & Dormann, 2018a) to address spatial bias in occurrence data. In this method, other environmental variables, used as bias covariates, characterize potential sources of sampling bias. Although this method is confirmed to be useful when dealing with sparse datasets (El‐Gabbas & Dormann, 2018b), it is highly dependent on the selected bias variable (Chauvier et al., 2021) that, in turn, intensifies upstream uncertainty caused by assisting covariates.

It is worth mentioning that bias adjustment and model parameterization depending on the prevalence of the target species result in varying spatial predictions (Araújo et al., 2019; Pottier et al., 2013). This variation is most noticeable for common species, and thus, distribution models of rare species that are habitat specialists may not be very sensitive to spatially‐biased occurrence data (Stolar & Nielsen, 2015). Being limited to a narrow gradient of environmental conditions, specialist species are thus more predictable and more distinguishable, i.e., high values of AUC of their SDMs, because of the high distinctiveness between their occurrence points and background space. In our case, this tendency was more obvious where narrow‐ranged mountain vipers (Ahmadi et al., 2019) obtained high scores of AUC and TSS. This, to a high extent, justify our SDM approach where, due to the sparse data of the mountain vipers, their occurrence points were pooled into one set. Since they belong to distinct taxonomic levels, e.g., species or sub‐species levels, the resulted SDMs might challenge niche equilibrium assumption (Wiens et al., 2009) and be prone to an inflated niche breadth (Pearman et al., 2010) where the resulted distribution models show higher levels of over‐estimation. However, niche inflation is more challenging for general species with abundant data (Randin et al., 2006). Moreover, the narrow‐ranged mountain vipers, in general, and the species/sub‐species belonging to the Raddei clade, in specific, show low rates of niche evolution and high degrees of niche conservatism (Ahmadi et al., 2021) that leads to the occupation of similar ecological conditions in these species.

## 5 CONCLUSION

Spatial bias of the input data is one of the main sources of uncertainty in the species distribution modeling approaches. This issue is particularly important for scarce species with geographically imbalanced‐biased data on their distribution ranges. Despite the great emphasis on the importance of model tuning and input data manipulation in improving SDMs, the performance of different models in using such an approach has not received much attention. In this research, we evaluated the performance of four commonly‐used SDMs to predict imbalanced‐biased occurrence points based on two methods of background data selection including random and background weighting. Our result reveals that different models produced dissimilar results for two background selection schemes. Complex GBM and RF models, due to their interpolative conception, showed inefficiency in predicting test points, especially for the background weighting mode. The GLM over‐predicted presence areas due to its extrapolative nature. Despite of being a machine learning method, MaxEnt shows a comparable performance in predicting test points in two background selection schemes. The results of the present study emphasize the proficiency of MaxEnt model in generating reproducible comparisons particularly when the input data is being completed.

## AUTHOR CONTRIBUTIONS

**Mohsen Ahmadi:** Conceptualization (lead); data curation (lead); formal analysis (lead); investigation (lead); methodology (lead); supervision (equal); validation (equal); visualization (lead); writing – original draft (lead); writing – review and editing (lead). **Mahmoud‐Reza Hemami:** Conceptualization (supporting); formal analysis (supporting); investigation (supporting); methodology (supporting); supervision (equal); writing – original draft (supporting); writing – review and editing (supporting). **Mohammad Kaboli:** Conceptualization (supporting); data curation (equal); investigation (equal); methodology (supporting); resources (equal); supervision (equal); writing – original draft (supporting); writing – review and editing (supporting). **Farzin Shabani:** Conceptualization (supporting); investigation (supporting); methodology (supporting); writing – original draft (supporting); writing – review and editing (supporting).

## CONFLICTS OF INTEREST

The authors declare no conflict of interest.

## Data Availability

All data are available on Dryad at: https://doi.org/10.5061/dryad.h9w0vt4ng.
